# Opportunities and Obstacles Associated With Sequential Immune Reconstitution Therapy for Multiple Sclerosis: A Case Report

**DOI:** 10.3389/fneur.2021.664596

**Published:** 2021-12-08

**Authors:** Riccardo Garbo, Daniela Cutuli, Simone Lorenzut, Gian Luigi Gigli, Daniele Bagatto, Mariarosaria Valente

**Affiliations:** ^1^Clinical Neurology Unit, Department of Neurosciences, Santa Maria della Misericordia University Hospital, Udine, Italy; ^2^Neurology Unit, Department of Neurosciences, Santa Maria della Misericordia University Hospital, Udine, Italy; ^3^Department of Medical Area (DAME), University of Udine, Udine, Italy; ^4^Neuroradiology Unit, Department of Diagnostic Imaging, Santa Maria della Misericordia University Hospital, Udine, Italy

**Keywords:** multiple sclerosis, cladribine, alemtuzumab, immune reconstitution therapy, case report

## Abstract

Cladribine is an effective disease-modifying treatment for relapsing-remitting multiple sclerosis that acts as an immune reconstitution therapy and is administered in a pulsed manner. Despite its efficacy, severe disease reactivation early after treatment represents a serious clinical problem, and clear evidence to guide the management of such a situation is lacking. Here, we describe the case of a patient experiencing considerable disease activity during the 1st year after the initiation of cladribine treatment. The patient was switched to alemtuzumab and, therefore, received double immune reconstitution therapy. Data regarding this approach are lacking, and real-world observations may be of interest. Despite achieving good control of disease activity, we observed several serious infectious complications. Our results suggest that sequential immune reconstitution therapies may be effective; however, at the price of higher susceptibility to infections.

## Introduction

Cladribine and alemtuzumab have proven to be effective treatments for relapsing-remitting multiple sclerosis (RRMS), and both act as immune reconstitution therapies administered in a pulsed manner (1–3). Disease activity may occur early after the first course of treatment. However, this does not necessarily imply a treatment failure that requires further modifications to the treatment strategy. For this reason, drug response evaluation is generally performed at least a few months after the second drug course (4). Nevertheless, relevant disease activity early after a treatment course of one of these drugs may sometimes represent a serious clinical problem, potentially leading to permanent disability. In the CLARITY trial, interferon beta-1a rescue therapy was used (1). However, evidence of managing such a problem is scarce, subsequently leading to different clinical choices in a real-world setting (5). Here, we report a case of considerable ongoing disease activity after the first course of cladribine treatment, which was managed with alemtuzumab administration. Data regarding this sequence of therapies, which act through immune system depletion and reconstitution, are lacking, and real-world observations are, therefore, of interest. After alemtuzumab treatment, the patient achieved disease stability; however, several infectious complications were observed. This suggests that this sequential treatment strategy can be applied but warrants caution and careful monitoring.

## Case Description

Here, we report the case of a 42-year-old patient diagnosed with RRMS at the age of 24 years, which was treated with different disease-modifying therapies. In 2012, after 2 years of natalizumab treatment, the patient was switched to fingolimod because of the high risk of progressive multifocal leukoencephalopathy. The patient remained stable until March 2017, when MRI progression was observed followed by a clinical relapse during the subsequent year.

Considering the presence of relevant disease activity, after discussing possible alternatives with the patient and considering an anti-JC virus (JCV) antibody index of 3.72, in May 2018, fingolimod therapy was discontinued and 9 weeks later, after lymphocyte count (ALC) recovery, oral cladribine was started. The patient received 1.75 mg/kg of cladribine and completed the first treatment course. The expanded disability status scale (EDSS) score at therapy initiation was 2.0, the ALC was 1,380 cells/μl, and baseline control MRI did not show any new lesions or contrast enhancement.

**Figure 1:**
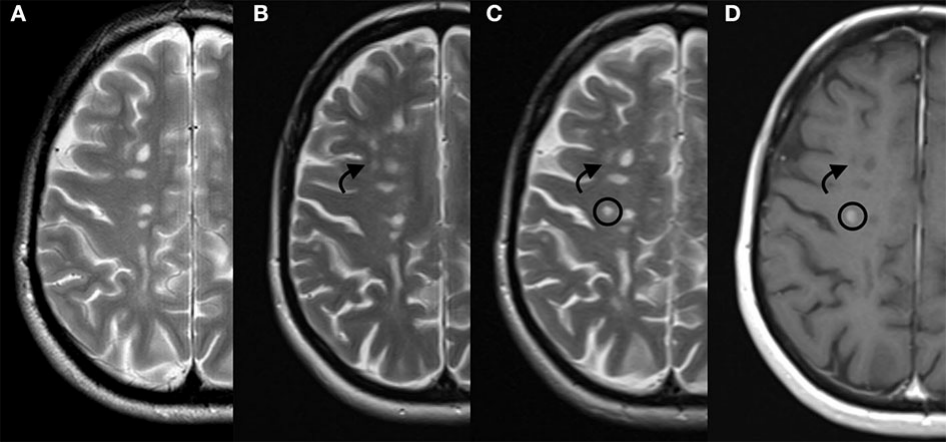
Patient's disease progression on MRI. From the left to the right: T2 weighted sequences respectively on June 2018 **(A)**, February 2019 **(B)**, and July 2019 **(C)**, and; contrast enhanced T1 weighted sequence on July 2019 **(D)**. Arrow indicates a new demyelinating lesion and circle indicates a new demyelinating lesion presenting contrast enhancement. MRI, magnetic resonance imaging.

The patient consulted us in February 2019, reporting a slowly progressive somatosensory symptomatology over the previous month, which was considered as a relapse with no impact on permanent disability. However, MRI was performed and revealed four new demyelinating lesions, two of which presented with contrast enhancement. In July 2019, a new MRI scan was obtained, revealing five new cerebral enhancing lesions (Figure 1). We decided to switch therapy from cladribine to alemtuzumab. Therefore, the patient did not receive the second course of cladribine. The first course of alemtuzumab was administered in September 2019, when the ALC returned to the normal range (1,060/μl). The patient received 200 mg oral acyclovir twice daily for a month after infusions.

After starting alemtuzumab, the patient did not present any clinical relapses, radiologic signs of disease activity, or worsening EDSS score until January 2021. Despite good disease control, the patient experienced various infectious complications. In November 2019, she was treated with oral amoxicillin/clavulanate to address an upper airway infection. In December 2019, the patient was hospitalized on a precautionary basis because of A/H3 influenza infection; however, she did not require treatment. At the end of January, she received oral antibiotic treatment for upper airway infection. In February 2020, the patient presented with dermatomal varicella zoster virus reactivation, which required hospitalization and intravenous acyclovir.

**Figure 2:**
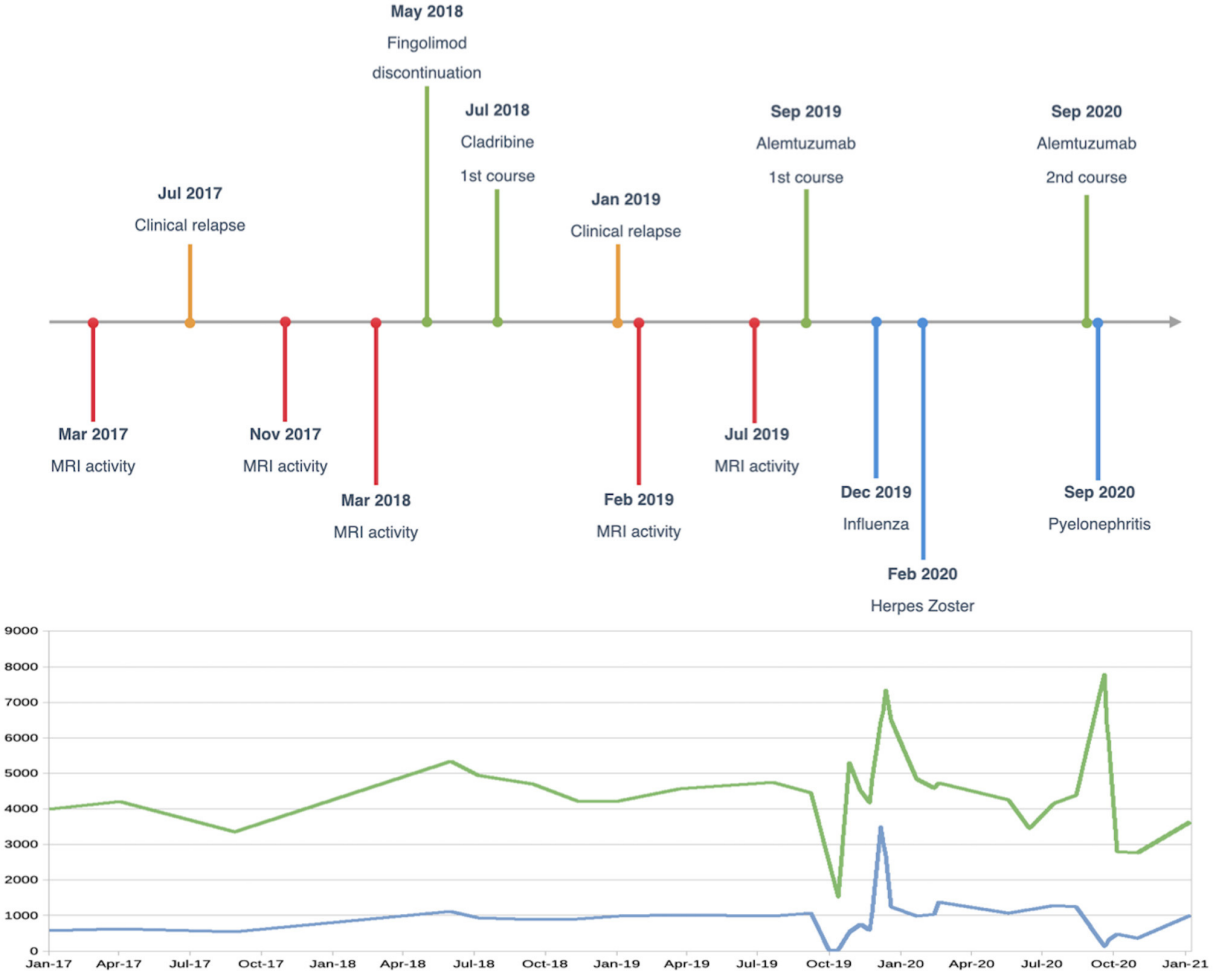
Case timeline, and white blood cell (green line) and lymphocyte (blue line) changes over time (cells/μl).

The second course of alemtuzumab was administered in September 2020. A few days after treatment, the patient was again hospitalized for *Escherichia coli-*related left pyelonephritis, with findings of a duplicated ureter, and was successfully treated with antibiotics. Case timeline is provided in Figure 2.

Written informed consent was obtained from the patient for the use of clinical data and imaging studies.

## Discussion

In the current report, we describe the management of ongoing disease activity in the 1st year after cladribine initiation in a patient previously treated with fingolimod. Although cladribine has been proved to be effective in highly active multiple sclerosis (6, 7), it might not be sufficient to control inflammatory activity after fingolimod withdrawal, as recently reported in other cases (8–10). Some indirect comparisons suggest that cladribine and fingolimod have similar efficacy (7, 11). However, it has been postulated that lymphocytes entrapped in the lymph nodes due to fingolimod action could evade depletion provoked by subsequent immune reconstitution therapies (9). In our case, cladribine was initiated only after ALC recovery.

Management of considerable disease activity that appears early after the administration of an immune reconstitution therapy course is challenging without strong evidence to guide clinical decisions. Regarding alemtuzumab, some cases of severe reactivation after the first treatment course have been described with different management strategies, including continuation of scheduled therapy (12) or administration of a B-cell depleting agent, such as rituximab (13, 14) or ocrelizumab (15). Both these strategies have been proven to be effective and safe. In more aggressive cases, autologous stem cell transplantation could be an option as well (16). Regarding cladribine, very scarce data are available in the literature, and switching to another highly effective therapy, as in our case, is thought to be a reasonable option (17). In the CLARITY trial, rescue therapy with interferon beta-1a could be applied for patients with highly active disease (1), and 2.5% of the patients in the cladribine 3.5 mg/kg group received this treatment (18). In the reported case, interferon therapy was not considered as the patient had already received it in the past, without successful disease activity control.

Treatment with natalizumab, fingolimod, rituximab, ocrelizumab, and autologous stem cell transplantation has also been reported in the 1st year after cladribine initiation, but without outcome details (5, 8, 9).

Both alemtuzumab and cladribine cause lymphocyte depletion. The extent of B cell reduction is quite similar among the two treatments, but with a slower repopulation rate under cladribine administration (19). Alemtuzumab provokes a more rapid lymphocyte depletion, has a broader degree of action, and causes a more profound and durable reduction of CD4+ and CD8+ T cells compared to cladribine (19). Unfortunately, since lymphocyte subset monitoring is not routinely required in clinical practice, we measured them only at a few time points. This makes these measurements of scarce interest, as no trend after treatments or correlation with disease activity or infectious complications could be identified. However, given the previously mentioned pharmacodynamics of these treatments along with other multiple sclerosis treatments, ALC may be of limited utility, and immunophenotyping may be helpful in guiding treatment decisions in the future.

Regarding efficacy, no head-to-head comparisons exist between cladribine and alemtuzumab, and the results from a network meta-analysis did not reveal any differences in the outcome measures (6). Longer follow-up will be required to assess the long-term efficacy of alemtuzumab in the reported case. However, breakthrough disease activity observed after cladribine initiation was rapidly and effectively controlled with the new subsequent immune reconstitution treatment. With this approach, there may be an augmented risk of side effects due to the additional action on the immune system. In trials of alemtuzumab, the more frequently observed infections included upper airway infections, influenza, herpetic virus infections, and urinary tract infections, as observed in our present case (20). Other opportunistic infections have been observed mostly within months after treatment initiation (21). In addition, an increased risk of herpes zoster infection has been reported in association with cladribine (18). Along with the infections reported during alemtuzumab treatment, an additional risk caused by previous cladribine exposure should also be considered in our patient. We waited for ALC normalization before alemtuzumab administration, but ALC was anyway lower than the levels observed before cladribine initiation. However, the status of ALC before an alemtuzumab treatment course does not predict any subsequent infection risk (20). Depletion of CD8+ T cells has been suggested to be associated with an increased risk of viral infection after alemtuzumab treatment (22). Although cladribine has a small effect on naive and memory CD8+ T cell counts, recovery at week 48 was minimal for naïve CD8+ T-cells and did not occur for memory CD8+ T cells in clinical trials (23). However, this aspect could be negligible considering the more profound T cell depletion induced by alemtuzumab.

In conclusion, alemtuzumab proved to be effective at controlling severe disease activity that appeared early after cladribine administration. However, the observation of different infectious complications warrants caution and a discussion about pharmacological prophylaxis for intercurrent infections. A longer follow-up and the description of similar cases may be helpful in the assessment of the efficacy and safety of sequential immune reconstitution therapies.

## Data Availability Statement

The original contributions presented in the study are included in the article/supplementary material, further inquiries can be directed to the corresponding author/s.

## Ethics Statement

Ethical review and approval was not required for the study on human participants in accordance with the local legislation and institutional requirements. The patients/participants provided their written informed consent to participate in this study. Written informed consent was obtained from the individual(s) for the publication of any potentially identifiable images or data included in this article.

## Author Contributions

RG: investigation and writing the original draft. DC: investigation, conceptualization, writing, reviewing, and editing. SL: conceptualization, writing, reviewing, and editing. GG: supervision, writing, reviewing, and editing, as well as resources. DB: visualization. MV: supervision, writing, reviewing, and editing. All authors contributed to the manuscript and approved the submitted version.

## Conflict of Interest

The authors declare that the research was conducted in the absence of any commercial or financial relationships that could be construed as a potential conflict of interest.

## Publisher's Note

All claims expressed in this article are solely those of the authors and do not necessarily represent those of their affiliated organizations, or those of the publisher, the editors and the reviewers. Any product that may be evaluated in this article, or claim that may be made by its manufacturer, is not guaranteed or endorsed by the publisher.
